# Impact of Lignin Content on the Properties of Hemicellulose Hydrogels

**DOI:** 10.3390/polym11010035

**Published:** 2018-12-27

**Authors:** Basel Al-Rudainy, Mats Galbe, Monica Arcos Hernandez, Patric Jannasch, Ola Wallberg

**Affiliations:** 1 Department of Chemical Engineering, Lund University, P.O. Box 124, SE-221 00 Lund, Sweden; basel.al-rudainy@chemeng.lth.se (B.A.-R.); mats.galbe@chemeng.lth.se (M.G.); 2 Department of Chemistry, Polymer, and Materials Chemistry, Lund University, P.O. Box 124, SE-221 00 Lund, Sweden; monica.arcos_hernandez@chem.lu.se (M.A.H.); patric.jannasch@chem.lu.se (P.J.)

**Keywords:** galactoglucomannan, lignin, lignin-carbohydrate complex, ultrafiltration, precipitation, hydrogel

## Abstract

Hemicellulose is a promising renewable raw material for the production of hydrogels. This polysaccharide exists in large amounts in various waste streams, in which they are usually impure and heavily diluted. Several downstream processing methods can be combined to concentrate and purify the hemicellulose. However, such an approach can be costly; hence, the effect of impurities on the formation and properties of hydrogels must be determined. Lignin usually exists in these waste streams as a major impurity that is also difficult to separate. This compound can darken hydrogels and decrease their swellability and reactivity, as shown in many studies. Other properties and effects of lignin impurities are equally important for the end application of hydrogels and the overall process economy. In this work, we examined the feasibility of producing hydrogels from hemicelluloses that originated from sodium-based spent sulfite liquor. A combination of membrane filtration and anti-solvent precipitation was used to extract and purify various components. The influence of the purity of hemicellulose and the addition of lignosulfonates (emulated impurities in the downstream processing) to the crosslinking reaction mixture on the mechanical, thermal, and chemical properties of hydrogels was determined.

## 1. Introduction

Hydrogels are hydrophilic networks of polymers that are commonly produced by crosslinking various types of synthetic polymers or polysaccharides [1]. The properties of the hydrogels that are produced today are shaped by the type of crosslinker, the inherent nature of the main polymer, and the process with which these hydrogels are produced. Synthetic polymers yield hydrogels with excellent water absorption, strength, and durability. However, the precursors to these synthetic polymers are petroleum-based which makes the products lacking in terms of sustainability.

Polysaccharides and natural polymers are alternatives to synthetic polymers, especially in the development of hydrogels for medical applications, for which biocompatibility and biodegradability are paramount [2]. Many hydrogel products have been synthesized using polysaccharides as raw materials, such as starch, dextran, alginate, cellulose, and chitosan [2,3,4,5,6].

Hemicellulose is a promising polysaccharide for the production of hydrogels. Its high abundance (constituting over 20% of wood cell walls) and current losses in pulp and paper waste streams (incinerated in recovery boilers) make this work important industrially and economically [7]. Hydrogels from hemicellulose with similar properties as synthetic polymer-based hydrogels have been produced by Söderqvist Lindblad, et al. [8], who showed that it is possible to replace fossil-based polymers with renewable waste material. However, the hemicellulose that was used was derived from steam explosion of spruce chips and did not represent the readily available hemicelluloses that are found in other process water streams.

Maleki, et al. [9] produced hydrogels from hemicellulose (galactoglucomannan) that was derived from thermomechanical pulping process water of spruce chips. The resulting single-network hydrogels were weak, as evidenced by their low shear modulus, which improved after a secondary crosslinking reaction. Consequently, the swelling ratio declined, but the swelling rate increased. Hemicellulose that has been derived from sodium-based spent sulfite liquor is another promising raw material for hydrogel production [10]—the product has swelling equilibria of between 50 and 270 g/g, a desirable property for use as absorbents and in agriculture.

All of the hemicelluloses [in this work, galactoglucomannan (GGM)] in the process waters above have at least one common problem: they exist in impure and highly diluted solutions (total dry substance of 5–6 g/L) and thus can not be used directly without being subjected to a proper concentration and purification method. These challenges have been addressed by other researchers using various types of downstream processing methods, such as membrane filtration, precipitation, and preparative chromatography [11,12,13,14,15].

Membrane filtration is effective in concentrating and, in certain cases, even purifying dilute streams. Al Manasrah, et al. [14] recovered 70% of the GGM in pressurized hot water extracts of spruce sawdust by ultrafiltration and reached a purity of 63%. Thuvander and Jönsson [16] reported that it was possible to separate lignin and GGM from thermomechanical process water using a combination of microfiltration and ultrafiltration. However, in other cases, this separation was difficult, due to membrane fouling [13,16,17]. Thus, membrane filtration was sufficient for concentrating and removing pulping chemicals, but another method was needed to separate lignin and GGM. 

In cases in which the separation of GGM and lignin is difficult, anti-solvent precipitation has proven to be an efficient method for separation after membrane filtration [11,15,18]. Zasadowski, et al. [11] precipitated GGM from TMP process water using acetone, obtaining a yield of 77% at an acetone concentration of 54.5%. Song, et al. [15] used ethanol as an anti-solvent to recover GGM from hot water extractives from spruce, achieving a yield of 78% at 90% ethanol and discovering that the precipitate contained solely polysaccharides with molecular weights that exceeded 4 kDa. 

Al-Rudainy, et al. [18] combined membrane filtration and anti-solvent precipitation for softwood spent sulfite liquor. Their GGM yield was on the same order of magnitude as those of other groups but with less anti-solvent, as a result of the membrane filtration step. Economically, it is preferable that the separation occurs in one step without the addition of any chemicals, such as anti-solvents. This tactic, however, will decrease the purity of the GGM due to the higher amounts of lignin in the concentrated process water.

The effect of additional lignin on the formation and properties of various products has been studied in other fields, such as in enzymatic treatment, wherein lignin has inhibitory effects on the reactions [19]. With regard to hydrogel production, Maleki, et al. [10] compared 2 hydrogels that were produced using the same methods and the same hemicellulose and but from 2 sources: spent sulfite liquor using ethanol precipitation and centrifugation as the separation and purification step versus a side stream of the steam explosion of spruce wood chips. The solution was concentrated by ultrafiltration and purified through diafiltration. As a result, the second specimen contained more lignin. This led to darker hydrogels with lower reactivity and swellability, which were theorized to have been caused by hydrophobic groups on the lignin, reducing the overall hydrophilicity of the hydrogel. However, the other differences between the 2 hemicelluloses have not been examined since.

In this work, a one-pot reaction was chosen to study the effects of lignin impurities on the properties of GGM hydrogel. Epichlorohydrin (ECH) was used as the crosslinker, and the GGM and lignin were acquired by membrane filtration and anti-solvent precipitation of softwood spent sulfite liquor.

## 2. Materials and Methods 

### 2.1. Raw Material and Reactants

For the raw material, sodium-based spent sulfite liquor was used, which was extracted from the first step in a 2-step softwood (60% *Picea abies* and 40% *Pinus sylvestris*) pulping process by Domsjö Fabriker (Örnsköldsvik, Sweden). The SSL contained 6.6% galactoglucomannan, 1.8% xylan, 0.8% arabinan, and 36.4% lignin, with a total dry substance of 84.6 g/L [17]. The remaining components were pulping chemicals, monosaccharides, acid-insoluble solids, and extractives.

The reactants that were used for the crosslinking reaction were 99% pure synthesis-grade epichlorohydrin and 50 wt % analytical-grade sodium hydroxide solution (Merck KGaA, Darmstadt, Germany).

### 2.2. Ultrafiltration and Anti-solvent Precipitation

The product was concentrated and low-molecular-weight components (such as pulping chemicals and monosaccharides) were removed by diafiltration per the membrane filtration methods in a previous study [17]. A 400-mL stirred filtration vessel that was equipped with a 50-kDa membrane (GR51PP, Alfa Laval Corp. AB, Lund, Sweden) and a 10-L feed tank were used for the membrane filtration setup. The pressure was monitored with a digital pressure gauge (DCS40.0AR, Trafag AG, Bubikon, Switzerland) and adjusted using a valve that was connected to a 6-bar nitrogen gas line. The crossflow velocity and temperature were controlled using a magnetic stirrer with a heating plate (MR2002, Heidolph Instruments GmbH & Co. KG, Schwabach, Germany), on top of which the 400-mL vessel was placed. The raw material was concentrated at 50 °C, a cross-flow velocity of 0.5 m/s, and 5.5 bar transmembrane pressure, decreasing in volume by 90% in fed batch mode. Diafiltration was then performed on the retentate to a diafiltration factor of 5 using deionized water in the feed tank. The membrane was washed with 0.04 wt % acid detergent solution (Ultrasil 73, Ecolab AB, Älvsjö, Sweden) at 50 °C for 1 h before and after each experiment. This procedure was repeated until the total weight of diafiltrated retentate exceeded 1 kg.

Precipitation was performed on 1 kg of diafiltrated retentate in a glass beaker with magnetic stirring (500 RPM) at room temperature, with acetone (SupraSolv MS, Merck Schuchardt OHG, Hohenbrunn, Germany) added gradually to a final concentration of 45 wt % [18]. The mixture was agitated for 15 min before being transferred to 750-mL centrifuge bottles (Beckman Coulter, Brea, California, USA) and was then centrifuged at 4000 RPM for 20 min (Jouan S.A., Model C412, Saint-herblain, Nantes, France). The liquid phase was carefully decanted, and the bottles that contained the precipitate (most of the galactoglucomannan) were dried in an oven at 50 °C for 48 h. The liquid phase (most of the lignin) was dried in a vacuum evaporator (BÜCHI Rotavapor R-153, BÜCHI Labortechnik AG, Flawil, Switzerland) at 50 °C and an average absolute pressure of 200 mbar for 60 h. Both powders were re-dissolved in deionized water (2 g powder to 18 g water) to make the stock solutions that were to be used in the hydrogel synthesis steps.

### 2.3. Preparing Hydrogels

Hydrogels were synthesized per reference [20]. A total of 2 mL stock solution (galactoglucomannan, lignin, or a mixture of both) was pipetted into a 3-mL vial, and 250 µL 50% sodium hydroxide solution was added and mixed for 5 min on a vortex mixer (MS2 Minishaker, IKA®-Werke GmbH & Co. KG, Staufen, Germany). The crosslinking reaction was then started by adding 100–300 µL epichlorohydrin and mixing it until the solution appeared to be homogeneous. Approximately 1 mL of the mixture was then transferred to a mold (flexible plastic bottle caps), covered, and left at room temperature for 48 h. The hydrogel was peeled off carefully from the mold and placed in 50 mL deionized water at room temperature for 24 h; the wash solution was then transferred to a container, and the same procedure was repeated to properly wash away any unbound reactants. The wash solution that was collected (approximately 100 mL) was later analyzed for lignin, hemicellulose, acetic acid, and glycerol content and epichlorohydrin conversion. 

### 2.4. Analysis

#### 2.4.1. Swelling Capacity

Swelling capacity was measured by carefully patting the newly prepared and washed hydrogel (Section 2.3) dry with tissue paper and weighing it. After the hydrogel was dried at 50 °C for 24 h, it was reweighed, and the swelling capacity (SC) was calculated per the following equation:

(1) $$\mathrm{SC}=\;\frac{m_{\mathrm{wet}}-m_{\mathrm{dry}}}{m_{\mathrm{dry}}}$$

where *m*_wet_ and *m*_dry_ are the mass of the hydrogel in the wet and dry states, respectively.

#### 2.4.2. Lignin Content and Epichlorohydrin Conversion

The concentration of lignin in solution was determined on a UV spectrophotometer (Shimadzu UV-visible spectrophotometer UV-1800, Kyoto, Japan). The wavelength was set to 234 nm, and the extinction coefficient was 31.6 L/g·cm [17].

Epichlorohydrin conversion was measured using chloride titrator strips (Quantab Chloride Low Range titrators, HACH company, Loveland, CO, USA). Chloride concentration was determined for the hydrogel wash solution, which, after the conversion, was calculated as the amount of chloride ions in the wash solution divided by the amount of chloride in the epichlorohydrin that was added. 

#### 2.4.3. Hemicellulose, Acetic Acid, and Glycerol Content

The hemicellulose concentration and glycerol content were determined per previous studies [18]. Ten milliliters of a sample were acid-hydrolyzed using 0.75 mL 72% sulfuric acid solution and autoclaved (Systec DX 150, Wettenberg, Germany) at 121 °C for 1 h. The hydrolysate was then vacuum-filtered to remove acid-insoluble solids and analyzed for monosaccharide content on a HPAEC-PAD system (ICS-3000, Dionex Corp., Sunnyvale, CA, USA) with a CarboPac PA1 carbohydrate analytical column. Deionized water was used as the eluent at a flow rate of 1 mL/min and 0.5 mL/min 200 mM sodium hydroxide postcolumn addition. The injection volume was 10 µL, and the hemicellulose concentration was calculated by summing the monosaccharide content and anhydro corrections of 0.88 for pentoses and 0.90 for hexoses. 

The acetic acid and glycerol content was determined on an HPLC system (LC-20AT, SIL-20AC, SCL-10A vp, RID-10A, CTO-20AC, Schimadzu Corp., Kyoto, Japan) with an Aminex HPX-87H column (Bio-Rad, Hercules, CA, USA), 5 mM sulfuric acid solution as eluent at a flow rate of 0.5 mL/min, a column temperature of 50 °C, and sample injection volume of 10 µL.

#### 2.4.4. Size-exclusion Chromatography

The molecular weight distribution was examined in liquid samples on a Waters size-exclusion chromatography system (Waters, Milford, MA, USA), with a Waters 600E system controller, coupled with a refractive index detector (Waters 2414 Differential Refractometer), a UV detector (Waters 486 Tunable Absorbance Detector) that was set to 234 nm, and a pump (Waters 600 gradient pump). Samples were injected using an autosampler (Waters 717 plus autosampler) with an injection volume of 20 µL. Fractionation was performed on a TSKgel column (G4000PWXL, TOSOH Bioscience GmbH, Griescheim, Germany), with deionized water as the eluent at a flow rate of 0.5 mL/min. Polyethylene glycol standards (Merck Schuchardt OHG, Germany) were used for the column calibration (100 kDa, 35 kDa, 10 kDa, 4 kDa, 400 Da).

#### 2.4.5. Fourier Transform Infrared Spectroscopy

The samples that were to be measured were dried at 50 °C in a furnace (Heraeus, Heraeus Holding GmbH, Hanau, Germany) and ground to a fine powder using a mortar and pestle. The pulverized samples were then measured with an FTIR (Bruker ALPHA-p FTIR spectrometer, Billerica, MA, USA) in attenuated total reflectance mode, in the wavenumber range of 4000 to 500 cm^−1^ with 2 cm^−1^ resolution and 72 scans per sample.

#### 2.4.6. Compression Stress and Strain

Compression tests were performed on newly prepared and washed cylindrical hydrogels. The hydrogels were prepared per Section 2.3, except that the mold was a 3-mL vial. Compression stress and strain were measured using a scale (PL6001-l, Mettler Toledo Inc., Columbus, OH, USA) and motor-controlled piston, for which the rate of axial displacement was 1.0 mm/min. Given that the axial displacement was constant, the strain was calculated by multiplying the elapsed time with the axial displacement rate. Stress was calculated as the mass that was displayed on the scale divided by the cross-sectional area of the hydrogel. 

#### 2.4.7. Thermogravimetric Analysis

Thermogravimetric analysis was conducted on a Q500 TGA (TA Instruments Inc., New Castle, DE, USA). Powdered samples were predried in a furnace at 50 °C for 24 h and cooled to room temperature using a desiccator prior to analysis. The samples were placed in open aluminum trays (2 to 5 mg) and heated from 15 to 600 °C at 10 °C/min under a nitrogen atmosphere (60 mL/min).

#### 2.4.8. Release of BTB from Hydrogels

Hydrogels were prepared as in Section 2.3, except that 4 mg of bromothymol blue powder (Merck Schuchardt OHG, Hohenbrunn, Germany) was added to the reaction mixture and dissolved by mixing prior to the addition of ECH. After 48 h, 2 hydrogel disks were carefully peeled off and placed into a mesh basket, which was then submerged into a beaker with 400 mL 4 g/L NaOH solution. The solution was then stirred magnetically (rod length of 2.5 cm) at 100 RPM, and 2-mL samples were taken at timed intervals to determine BTB concentrations.

The BTB concentration was measured on a UV spectrophotometer (Shimadzu UV-visible spectrophotometer UV-1800, Kyoto, Japan) at the isosbestic point (wavelength 498 nm). The extinction coefficient was 9.31 L/g·cm, which was calibrated using various concentrations of BTB in 4 g/L sodium hydroxide solution.

#### 2.4.9. Liquid Chromatography-mass Spectrometry (LC-MS)

The LC instrument was part of the 1260 Infinity II line (Agilent Technologies, Waldbronn, Germany) and comprised a range of modules: a Quaternary Pump VL (G7111A), a Vialsampler (G7129A), and a temperature-controlled column compartment (G7130A). The column, a 4.6 mm × 100 mm Poroshell 120 EC-ECS, was operated at 50 °C. The eluent flow rate was 0.5 mL/min, with a gradient switch between eluents A (0.1% formic acid solution) and B (95% acetonitrile in 0.1% formic acid solution) according to the program in Table 1. The injection volume was 1 µL, and the total run time per sample was 12 min. LC detection was performed using a UV diode array detector (G7115A) with a peak width of 0.1 min and a 4-nm slit.

The LC system was connected to a mass spectrometer (G6545B Q-TOF) that was equipped with a dual AJS ESI ion source that was operated in ESI positive mode. The nitrogen gas temperature was set to 350 °C at a flow rate of 12 L/min. The nebulizer pressure was 35 psig, with a sheath gas temperature and flow rate of 400 °C and 12 L/min, respectively. The nozzle voltage was 1000 V, the collision energy was set to 0 eV, and the data were acquired in the range of 50 to 3200 *m*/*z*.

## 3. Results and discussion

### 3.1. Ultrafiltration and Anti-solvent Precipitation

Table 2 summarizes the results from the re-dissolved fractions after ultrafiltration and anti-solvent precipitation. The separation method clearly fractionated the spent sulfite liquor components into 2 major fractions: a polysaccharide-rich stream and another that contained primarily lignin. 

**Figure 1:**
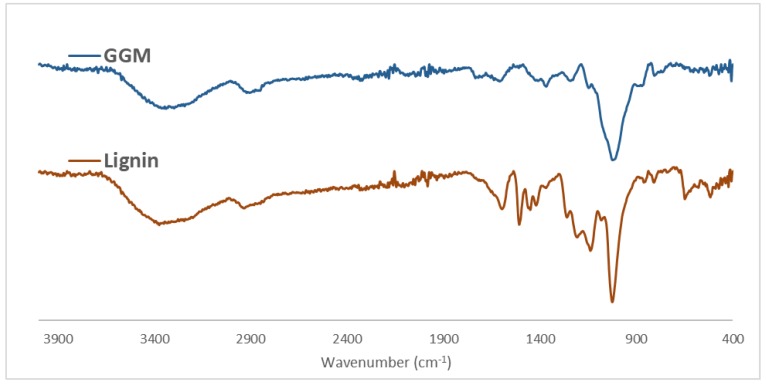
FTIR spectrum comparison of the GGM and lignin fraction.

The difference in properties of these fractions was also observed using FTIR spectroscopy (Figure 1). In the region between 3000–3500 cm^−1^, a broad signal appeared in both fractions and was assigned to the stretching of O–H bonds. The peaks at 2930 and 2852 cm^−1^ were caused by the stretching of C–H bonds in the lignin and polysaccharides [21,22]. The peak at 1736 cm^−1^ was assigned to the acetyl groups on the GGM and was visible in both fractions, albeit at greater intensity in the polysaccharide-rich fraction. Various peaks from aromatic skeletal vibrations appeared in the lignin-rich fraction (1602, 1509, 1451, and 1420 cm^−1^). These peaks were missing or lower in intensity in the polysaccharide fraction, indicating proper separation between these entities. Other indicators of good separation were the peaks for various guaiacyl moieties (1262–1142 cm^−1^), which also implicated the lignin as being guaiacyl-type, as shown in our previous study [18].

The signals at 1082 and 1026 cm^−1^ were ascribed to vibrations of asymmetrical C–O–C bonds. The signal at 1082 cm^−1^ was less intense in the lignin-rich fraction, indicating that it originated from internal vibrations of the pyranose structure in the polysaccharides [21]. Two signals were detected in the low wavenumber region and were assigned to the sulfonate groups in the lignin (650 and 517 cm^−1^).

**Figure 2:**
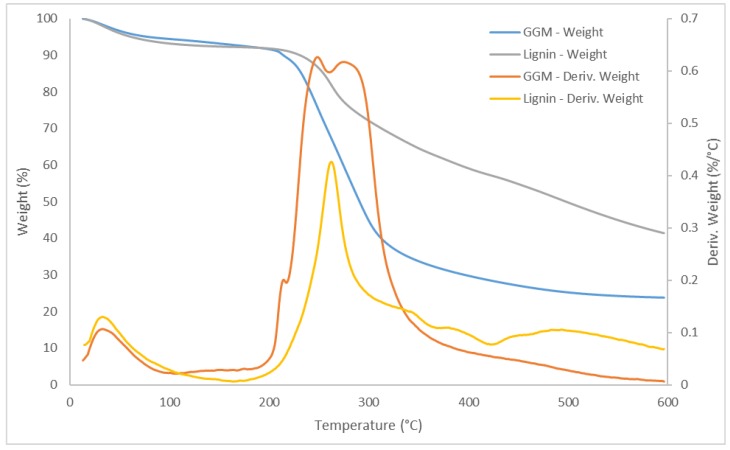
TGA and DTG curves for the GGM and lignin fraction.

The properties of various components can also be characterized using TGA, as applied to our 2 fractions; the results are presented in Figure 2. The degradation of lignin occurred in 3 major stages, as indicated by the derivative of the TGA curve (DTG). In the first stage, the DTG peak temperature was approximately 33 °C, due to the release of absorbed water and other solvents, such as acetone, that were used during the precipitation [23,24].

In the second stage, which had a DTG peak temperature of 263 °C, residual hemicelluloses were degraded, perhaps as were lignin-carbohydrate complexes. Certain lignosulfonates begin to degrade at temperatures above 250 °C [24,25]. Because the observed mass of the second-stage peak (~20 wt %) was higher than the total amount of hemicelluloses (Table 2), this result was expected as a possibility, even in our case. 

In the third stage (temperatures above 300 °C), several DTG peaks were observed. The visible peaks appeared at 344, 390, 441 and 493 °C, corresponding to the pyrolytic degradation of lignin; the release of phenolic derivatives, mercaptans, SO_2_, CO, and CO_2_; and the decomposition of aromatic rings at temperatures exceeding 500 °C [23]. The residual mass of the lignin-rich fraction at 600 °C was roughly 41 wt %. This high char residue content was anticipated, given that the lignin was sulfonated. According to previous reports [25,26], the remaining chemically bonded sulfonate groups and sodium salts were responsible for the residue and require temperatures above 600 °C for further decomposition. 

GGM decomposed in 2 major stages, the first of which was identical to the first stage of lignin decomposition. In the second stage, at least 3 DTG peaks could be observed at 215, 248, and 274 °C, with a residue of 24 wt % at the end of the run. The first DTG peak of the second stage had a mass that corresponded to 3.2 wt % of the total sample, similar to the amount of acetic acid (acetyl groups) in the sample per Table 2, indicating that the acetyl groups were the first components to leave the matrix, as shown by others [27]. The next DTG peak arose at 248 °C, near the reported temperature of the maximum rate of weight loss for softwood xylan [27]. However, the mass of the peak exceeded the amount of xylan that was available in the sample, suggesting that other compounds were decomposing at approximately the same temperature—likely the highly branched GGM, which has been reported to have a low decomposition temperature compared with low-branched linear GGM. 

The last DTG peak (274 °C) most likely contained the remaining GGM but also other galactan-based polysaccharides, as reported by Beall [27]. Overall, these results show that the lignin fraction is thermally more stable than the polysaccharide-rich fraction in terms of the temperature at the maximum rate of weight loss and the slower decomposition rate of the lignin fraction, as seen from the slope of the TGA curve.

### 3.2. Parameter Study

**Figure 3:**
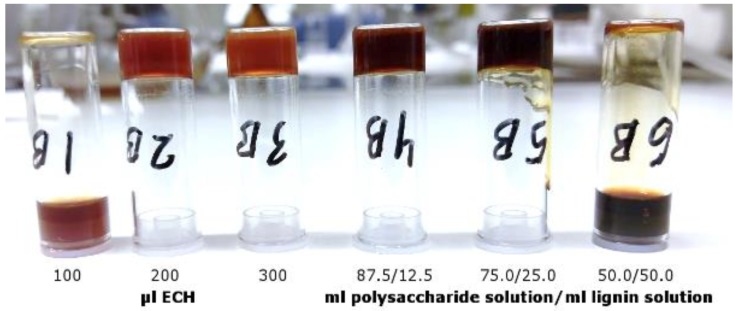
Visual representation of the resulting hydrogels after crosslinking of the polysaccharide solution (1B, 2B and 3B) at different ECH additions. The vials 4B to 6B show the effect of different lignin additions at a constant ECH dosage of 200 µL.

To determine the amounts of crosslinker and lignin that were required to form a stable gel, we performed a parameter study, based on the results that were presented in another report [20]. The various gels are shown in Figure 3. Vials 1B, 2B, and 3B are the results for the polysaccharide-rich stock solution after crosslinking 2 mL of the solution with 100, 200, and 300 µL epichlorohydrin (ECH), respectively, and a fixed amount of sodium hydroxide. A stable gel was formed with 200 µL ECH and higher. Decreasing the amount of ECH to 100 µL failed to form a coherent gel, due to the low degree of crosslinking, leaving the polysaccharide matrix stable in solution [28].

The color of the hydrogels differed, depending on the degree of crosslinking. The color forms from the dissociation of phenolic groups in the residual lignin in the stock polysaccharide solution [29,30] and is an indirect indication of the amount of sodium hydroxide that is consumed in the reaction; thus, the crosslinking reaction progressed further with the higher versus lowest amount of ECH.

Vials 4B to 6B show the effect of increasing amounts of lignin in the reaction mixture. The total dry substance, ECH, and sodium hydroxide levels were kept constant. To emulate the effect of greater lignin impurity in the stock solution, the amount of lignin was changed by decreasing the ratio of polysaccharide to lignin stock solution. Vials 4B to 6B had volumetric ratios of 87.5/12.5, 75.0/25.0, and 50.0/50.0 mL polysaccharide solution/mL lignin solution, respectively. In the latter sample, no coherent hydrogel formed; thus, the lignin concentration below this threshold was chosen for further study.

### 3.3. Effect of Crosslinking on Hydrogel Content

**Figure 4:**
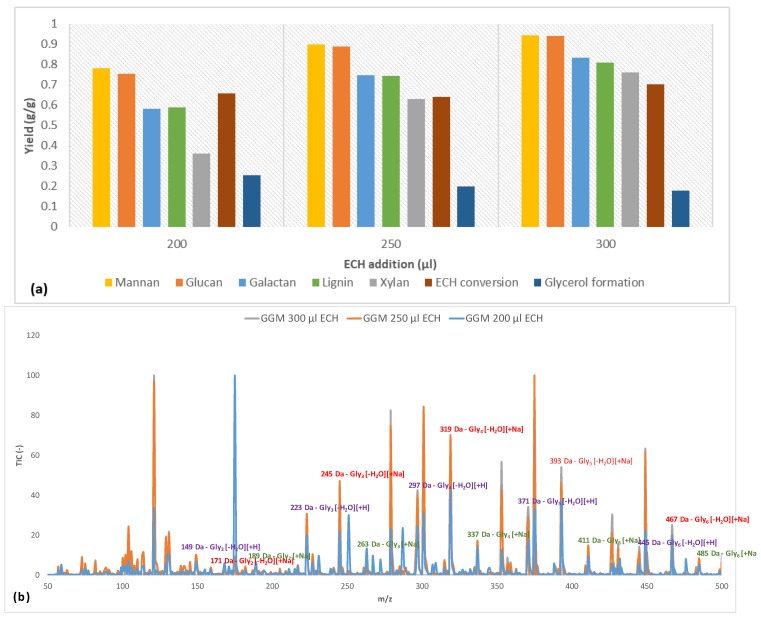
(**a**) The yields at different crosslinker additions for the various hydrogel bound solutes, ECH consumption and glycerol formation after the crosslinking reaction. (**b**) LC-MS of the wash solution (containing non-bound compounds) with highlighted *m*/*z* for the formed polyglycerols after the crosslinking reaction. Glyx is a polyglycerol with x number of subunits.

The parameters that were determined in Section 3.2 were applied to crosslink the polysaccharide-rich fraction with ECH volumes of 200, 250, and 300 μL. The results for the hydrogel content (percentage of component/total amount of component in the reaction mixture) are presented in Figure 4. 

Initially, the mannan and glucan yields were approximately on the same order of magnitude for the 3 amounts of crosslinker. This result was not surprising, because the main polysaccharide in the solution was galactoglucomannan [18]. However, the small difference that existed might have been due to some of the glucans being bound to other components. The yields of galactan followed the increases in mannan and glucan throughout the series but were lower overall, because there were other galactan-based polysaccharides in the solution that were not associated with GGM. The 2 most common such polysaccharides in the raw material are arabinogalactan and B-galactan [18]. Also, certain polysaccharides were bound to lignin and formed lignin-carbohydrate complexes (LCCs). A clear indication of the existence of these complexes is found in the figure, in which the galactan yield approximated that of lignin for all samples (Figure 4). Furthermore, the galactans in the solution have a higher molecular weight than GGM [18,31], demonstrating that the crosslinking reaction did not depend on MW in this case. Overall, the yield was high in terms of the utilization of solutes, reducing the potential waste after the reaction. 

The ECH conversion rate was between 64% and 70%; there was no discernable pattern versus the addition of ECH. However, the amount of ECH that was consumed increased as the proportion of ECH became larger, indicating that more glycerol bridges were formed between the polysaccharides (as evidenced by the rising yields in polysaccharides in Figure 4), but the formation of monoglycerol ether units could not be ruled out [32]. A common side reaction of crosslinking is the hydrolysis of ECH to glycerol, which in turn can react with ECH to form polyglycerols, as described by Kartha and Srivastava [32]. As shown in Figure 4, the formation of glycerol after the reaction decreased with higher ECH content. 

Earlier studies provide explanations for the phenomenon [32]. One reason is that a higher level of ECH shifted the reaction from homogeneous to heterogeneous due to the low solubility of ECH in water (roughly 7%), wherein the heterogeneous reaction of ECH has been found to result in low glycerol yields. Also, at an excess of ECH, the reaction may begin to polymerize ECH into dimers and trimers. Therefore, it is also possible that these polyglycerols form and bind to the hydrogels at higher amounts of ECH, thus lowering the observed glycerol yield. This phenomenon was also indicated by the LC-MS measurements, as seen in Figure 4b, in which glycerol polymers were found to contain up to 6 glycerol units. The large number of peaks that were detected was the result of various existing adducts (sodium and proton) and the dehydration of polyglycerols to varying extents [33]. However, the trend was the same, regardless of which adduct or dehydrated peak was compared. 

By comparing the signals with the strongest intensity (mono-dehydrated polyglycerols with sodium adduct), we found that the intensity for polyglycerols with over 4 glycerol units rose with increasing ECH levels. Notably, this finding was consistent with decreasing intensity in the region with fewer than 4 glycerol units for the sample with the highest amount of ECH, highlighting the transition from crosslinking monoglycerol units to oligomers. The overall trend in Figure 4b was a shift in the distribution of polyglycerol toward a higher degree of polymerization with increasing ECH levels, strengthening the observations made regarding the decrease in the formation of glycerol.

### 3.4. Effect of Addition of Lignin on Hydrogel Content

**Figure 5:**
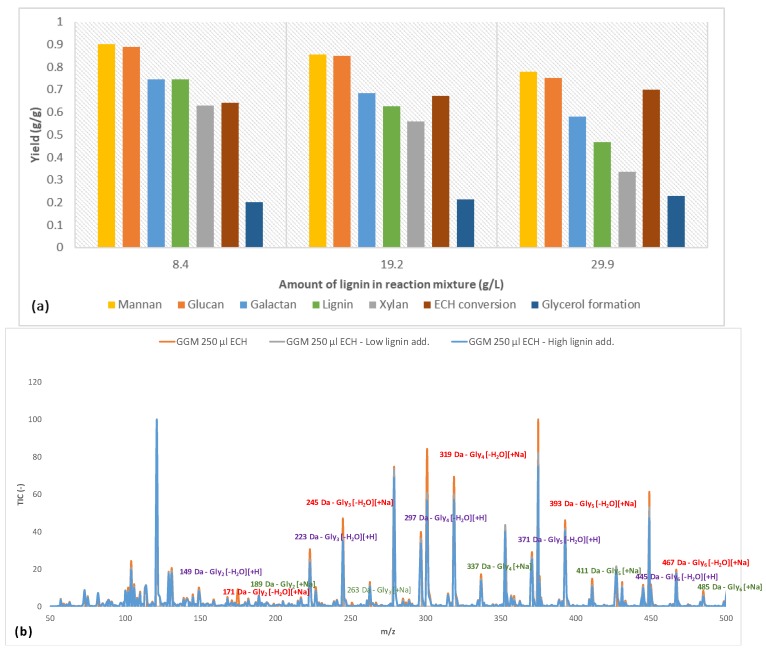
(**a**) The yields at different lignin additions for the various hydrogel bound solutes, ECH consumption and glycerol formation after the crosslinking reaction. (**b**) LC-MS of the wash solution (containing non-bound compounds) with highlighted *m*/*z* for the formed polyglycerols after the crosslinking reaction. Glyx is a polyglycerol with *x* number of subunits.

Based on the results of the parameter study (Section 3.2), various amounts of the lignin fraction were added to the reaction mixture. The data on the content of the synthesized hydrogels are presented in Figure 5. Adding lignin to the mixture decreased the yield for most solutes. Mannan and glucan continued to follow the same trend regarding yields, as shown in Section 3.3. The yields at high lignin content (29.9 g/L) were nearly identical to those for GGM hydrogels with low crosslinking (Figure 4), indicating that the crosslinking reaction favored the reaction with GGM, followed by galactans and xylans, independent of the lignin that was added. 

However, the yields for lignin differed from those in Figure 4. The trend was for more of the lignin to bind to the hydrogel, even with decreasing yield. These results implicate fewer crosslinks between the polysaccharides after the addition of lignin. Nevertheless, the consumption of ECH clearly rose, attributed to the formation of more glycerol (Figure 5a) but also perhaps to the crosslinking of low-MW non-gel-bound lignin. The polymerization of glycerol declined with increases in lignin addition by LC-MS (Figure 5b), supporting the possibility of the polymerization of low-MW non-gel-bound lignin.

### 3.5. FTIR on Hydrogels

**Figure 6:**
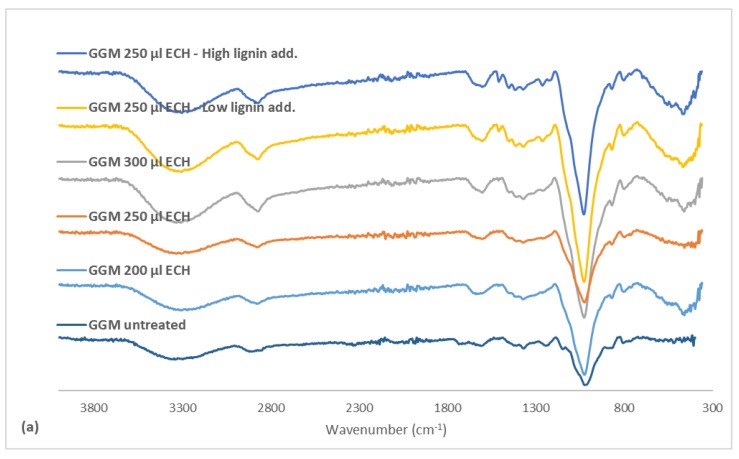

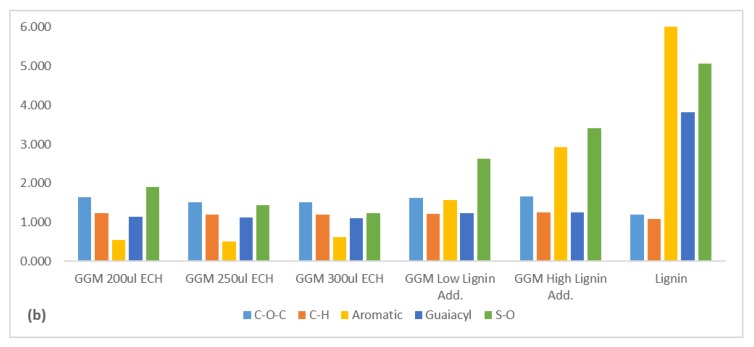
(**a**) FTIR spectrum comparing the produced hydrogels. (**b**) The ratio between the FTIR absorbance of a hydrogel and the non-treated GGM at different wavenumbers. C–O–C is the ratio for the ether bonds (1026 cm^−1)^, the C–H ratio was based on the wavenumber 2930cm^−1^, aromatic was measured at 1509 cm^−1^, guaiacyl was calculated as the sum of the three wavenumbers between 1262–1142 cm^−1^ and S–O absorbance was measured at 650 cm^−1^. The legend includes the ECH and lignin additions for the crosslinking reaction. Low lignin addition represented the 87.5/12.5 mL polysaccharide solution/mL lignin solution and the high lignin addition 75.0/25.0 mL polysaccharide solution/mL lignin solution.

FTIR measurements were performed on the hydrogels (see Figure 6a). The spectra for the hydrogels were nearly identical to that of the polysaccharide fraction, differing only by the disappearance of the peak at 1736 cm^−1^ (acetyl groups) for the hydrogels. Deacetylation usually occurs when the pH is higher than 3 and at high temperatures [34,35]. Further, at high sodium hydroxide concentrations, it could ensue at lower temperatures [20]. Given that the pH after the addition of sodium hydroxide to the reaction mixture was over 13, this latter mechanism was what likely occurred. 

No other differences were expected, because the major modification to the hydrogels was the addition of the glycerol bridge and the removal of hydroxyl groups, both of which had signals that coincided with those in the polysaccharide fraction. However, the intensities of these signals will vary, depending on the type of modification that occurred. These changes in intensities were seen by comparing the ratios of the absorbance peak height on various signals (Figure 6b). The gels had a higher ether bond (C-O-C) intensity than the polysaccharide fraction, which was expected, because crosslinking increases the formation of ether bonds and the removal of hydroxyl (OH) groups. However, the ratio decreased with rising ECH, which does not necessarily mean that the proportion of ECH that reacted declined overall. Yet, the proportion of crosslinks was smaller than that of hydroxyl groups, perhaps due to the formation of mono-glycerol ether units instead of a glycerol bridge, instead increasing the hydroxyl groups overall, in turn supporting the previous claim (Figure 4). 

The addition of lignin to the samples increased the C–O–C/OH ratio, likely due to the higher C–O–C/OH ratio in the added lignin. The strong indicators for bound lignin was the peak at 1509 (aromatic skeletal vibrations), the sum of the peaks for guaiacyl structures (1262–1142 cm^−1^), and the sulfonate groups (650 cm^−1^). The rise in crosslinking did not have a major impact on the ratios of the aromatic skeletal vibrations or the sum for the guaiacyl signals, indicating that the base structure of the lignin was consistent. However, the ratio for the sulfonate groups decreased. These results thus show that the highly charged (sulfonated) lignin was bound first, followed by the less sulfonated lignin. The increase in lignin before the crosslinking reaction enhanced the intensity of these peaks, wherein the sample that contained the highest amount of lignin had the strongest signals in this region. These results, coupled with the trend in Figure 5, thus show that some of the added lignin bonded to the hydrogel. 

### 3.6. Size-Exclusion Chromatography

**Figure 7:**
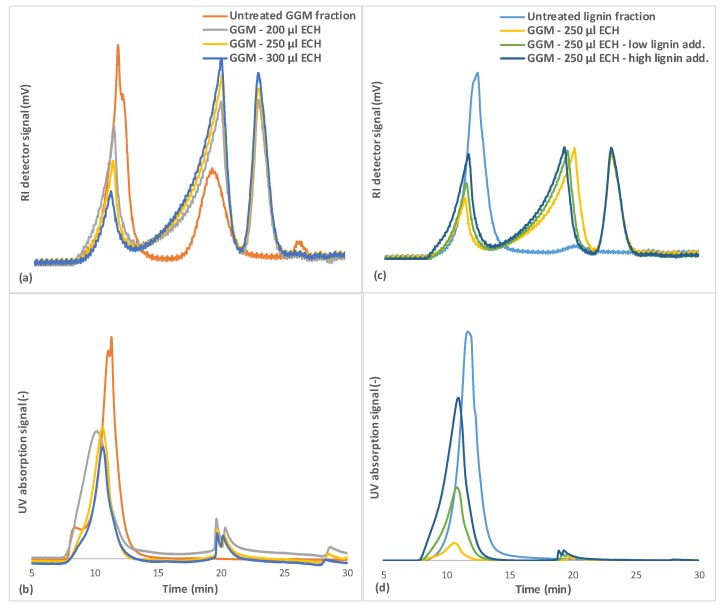
SEC for the untreated GGM and lignin solutions and the wash solutions after the crosslinking reaction. (**a**,**c**) is showing the response from the RI detector, and (**b**,**d**) the UV detector. The legend includes the ECH and lignin additions for the crosslinking reaction. Low lignin addition represented the 87.5/12.5 mL polysaccharide solution/mL lignin solution and the high lignin addition 75.0/25.0 mL polysaccharide solution/mL lignin solution.

The SEC results (Figure 7a) demonstrated that the GGM fraction contained 3 major MW regions. The high-MW region (between 5 and 10 min of retention time) contains the lignin and lignin-carbohydrate complexes [17,18], as evidenced by the UV absorption of that fraction (Figure 7b). The mid-MW region (approximately 20 min, Figure 7a) contained GGM and has little to no UV absorption; thus, it harbored low to negligible amounts of lignin. The third peak (>25 min) contained most of the remaining low-MW compounds, such as monosaccharides and pulping chemicals. 

The results in Figure 7a also show the MW of components that were not bound during the hydrogel synthesis (wash solution) at various degrees of crosslinking. The results implicated the mid-MW compounds being consumed first at low ECH concentrations, followed by the high-MW compounds, the consumption of which rose with the addition of ECH. The remaining peak in the mid-MW region was possibly lignin that was released from the high-MW lignin-carbohydrate complexes by hydrolysis, based on the increase in UV absorption in that region, as seen in Figure 7b and confirmed in our previous work [17], in which enzymatic hydrolysis of the carbohydrate segment of the LCCs released lignin in the same MW range. The same trend was seen for the case in which the lignin concentration increased in the reaction mixture (Figure 7c,d). 

Previous observations (Section 3.4 and Section 3.5) showed that not all of the lignin was bound to the hydrogel but that more ECH was consumed with increases in lignin, because the crosslinking of the lignin yielded larger water-soluble macromolecules that did not bind to the hydrogel. 

The rise in MW can be observed in Figure 7d, in which the MW of the lignin shifted toward the heavier end of the spectrum after the crosslinking reaction. The reason for the lignin failing to bind to the hydrogel could be the difference in solubility between these components. The solubility of GGM likely decreased after the addition of sodium hydroxide to the reaction mixture, due to the deacetylation of the polysaccharide, leading to heterogeneous (slow) crosslinking [36]. The solubilities of lignin and lignosulfonates during alkali conditions do not change (at room temperature), and thus, the crosslinking reaction is more homogeneous (fast). A third peak was observed after the crosslinking reaction in the region at approximately 23 min (Figure 7), likely comprising crosslinked monosaccharides and oligosaccharides or possibly polyglycerols, as identified by LC-MS in Figure 4b and Figure 5b.

### 3.7. Swelling Degree and Drug Release

The swelling behavior of the hydrogels was determined in deionized water. Swelling degree can be altered by changes in the amount of crosslinker or by the concentration of functional materials [10]. The swelling behavior was therefore expected to change, depending on the amount of ECH that was added but also possibly the lignin levels. 

**Figure 8:**
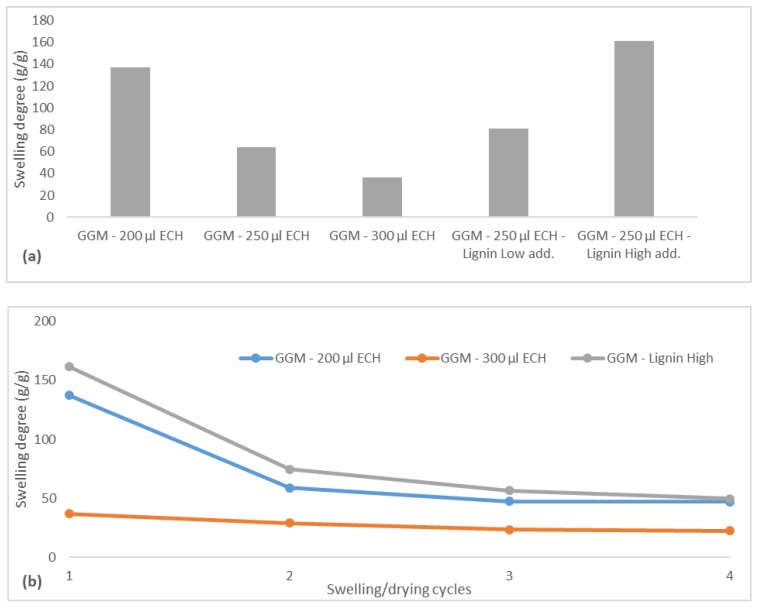
(**a**) The swelling degree for the different hydrogels after synthesis. (**b**) The swelling degree for three selected hydrogels after consecutive swelling and drying cycles. The legend includes the ECH and lignin additions for the crosslinking reaction. Low lignin addition represented the 87.5/12.5 mL polysaccharide solution/mL lignin solution and the high lignin addition 75.0/25.0 mL polysaccharide solution/mL lignin solution.

The results on the swelling degree of the hydrogels are presented in Figure 8a. The hydrogel with the lowest degree of crosslinking had an initial swelling degree of 137 g water/g dry hydrogel, which was in the same range as reported by Maleki, et al. [10] using the same raw material. As expected, the swelling degree of the hydrogels decreased with greater amounts of crosslinker, because the higher amount of crosslinks that are formed in turn compacts the hydrogel structure and decreases the number of available sites for water to penetrate [37,38]. At the highest level of ECH, the hydrogel had a swelling degree of approximately 38 g/g—much lower than the low-crosslinked hydrogel but still in the range of usable hydrogels for many applications. 

**Figure 9:**
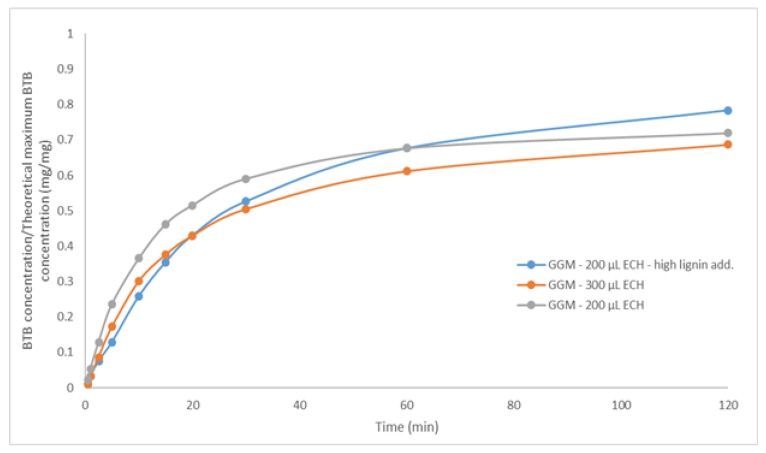
The release of BTB from three different hydrogels as a function of time. The legend includes the ECH and lignin additions for the crosslinking reaction. High lignin addition corresponds to 75.0/25.0 mL polysaccharide solution/mL lignin solution.

Increasing the amount of lignin in the hydrogels had the opposite effect, with the swelling degree instead rising, perhaps because the lignin consumed the ECH and remained soluble, yielding a hydrogel that was less crosslinked and had higher swellability, as indicated. Another explanation is that the addition of ionizable components (lignosulfonates) increased the initial osmotic pressure during swelling, thus resulting in a higher driving force for water uptake [39]. This phenomenon was observed when the BTB release was measured (Figure 9). By comparing the hydrogel with low and high ECH content, it was clear that the release of BTB was slower in the latter case, as expected, because the reduced mobility of the polymer impeded the penetration of the solvent [39]. The opposite effect was anticipated for the sample to which a higher amount of lignin was added, given that the swellability was greater (Figure 8a). However, the release was slower, as shown in Figure 9, likely due to the higher osmotic pressure and thus greater water influx at the beginning of the release of BTB. This effect diminished after approximately 30 min, at which point the high-lignin hydrogel was nearly completely swollen, and thus, the release of BTB increased.

The repeated swelling and drying of hydrogels is an important property for certain applications in which the reusability of hydrogels is a key element, such as in agriculture [10]. This step was performed for 3 of the hydrogels, as seen in Figure 8b. All hydrogels experienced the largest change at the beginning of the swelling-drying cycles and reached a constant swelling degree after 2 consecutive cycles. The high-lignin hydrogel and that with the low ECH reached a stable swelling degree of approximately 50 g/g, which was greater than for the highly crosslinked hydrogel (22 g/g). That these hydrogels reached the same equilibrium strengthens the observations in Figure 8a. 

### 3.8. Mechanical Strength

**Figure 10:**
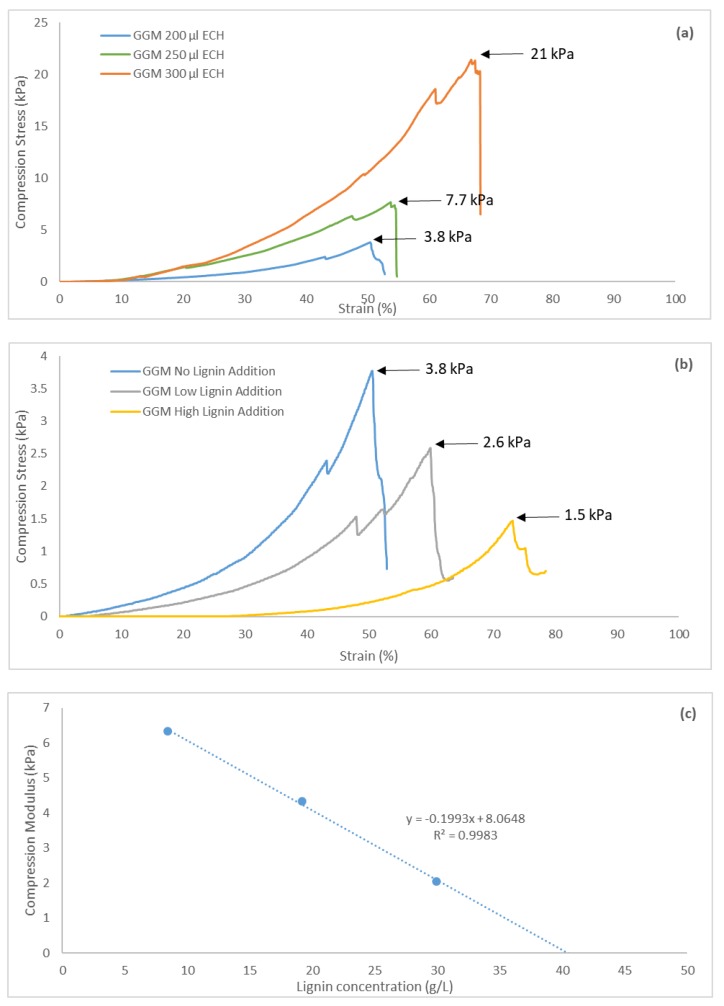
Compression stress and strain for (**a**) three hydrogels at different ECH additions and (**b**) three hydrogels with different lignin additions and a constant (200 µL) ECH addition. Low lignin addition represented the 87.5/12.5 mL polysaccharide solution/mL lignin solution and the high lignin addition 75.0/25.0 mL polysaccharide solution/mL lignin solution. (**c**) A curve of the compression modulus as a function of lignin concentration in the base reaction mixture. The compression modulus was determined at the point of maximum UTS.

The cylindrical hydrogels were tested mechanically to obtain compression stress-versus-strain curves. All of the hydrogels demonstrated strain tolerances of over 44%, as seen in Figure 10. The curves were not linear but followed an exponential increase, demonstrating the softness of the gels [40]. The maximum ultimate tensile strength (UTS) for these hydrogels was 21 kPa, which is in the same range as for other pure hemicellulose hydrogels [41]. The graphs also show that a yield strength existed wherein further compression of the hydrogels resulted in irreversible deformation, due to internal fractures or breaks, which were visible throughout the transparent hydrogel during compression. 

Greater crosslinking enhanced the UTS of the hydrogels, decreasing the strain (Figure 10a), thus yielding hydrogels with greater mechanical strength and less flexibility (higher brittleness). This finding was not apparent from the results because the strain also increased with the addition of ECH. However, the compression modulus—the ratio between compression stress and strain—rose with the addition of ECH; thus, the stiffness of the hydrogels increased [40]. The opposite effect was observed with higher lignin content (Figure 10b). The UTS of the hydrogels decreased with greater amounts of lignin, likely attributed to the fewer crosslinks that formed when lignin was added. The compression modulus also declined, indicating that the hydrogels had greater flexibility, likely caused by the higher swelling degree. 

During the parameter study, a limit for hydrogel formation was reached when 50/50 mL polysaccharide solution/mL lignin solution was added, corresponding to a near-zero compression modulus, based on the lack of a network structure that resisted the compressible forces [42]. A linear relationship was observed between compression modulus and lignin concentration in the bulk solution (Figure 10c). Extrapolation of the curve to a compression modulus of 0 gave the lignin a concentration of 40.5 g/L, which was well in the range of the limit that was observed during the parameter study. 

### 3.9. Thermo-gravimetric Analysis

**Figure 11:**
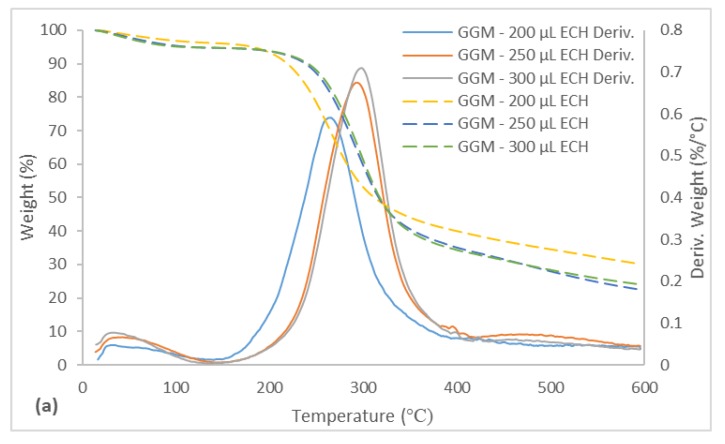

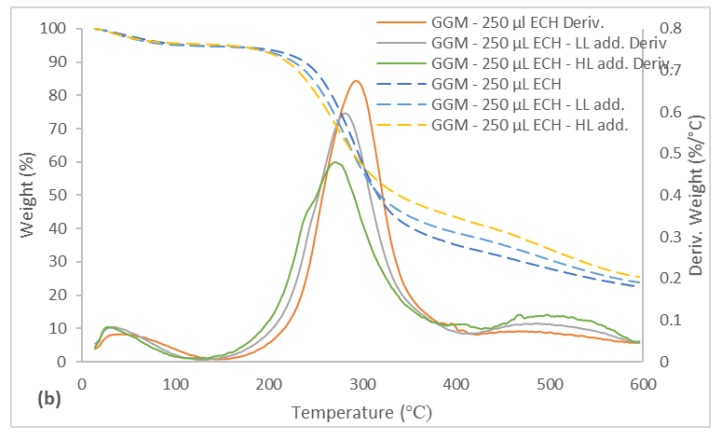
TGA and DTG curves for the synthesized hydrogels between the temperatures of 15 and 600 °C. (**a**) The legend includes the ECH and lignin additions for the crosslinking reaction. (**b**) (LL) Low lignin addition represented the 87.5/12.5 mL polysaccharide solution/mL lignin solution and (HL) the high lignin addition 75.0/25.0 mL polysaccharide solution/mL lignin solution. Deriv. is the acronym for the DTG curve.

TGA was performed on the synthesized hydrogels (Figure 11). All of the hydrogels had a DTG peak at approximately 33 °C, which corresponded to residual solvents, as seen with the purified GGM and lignin in Section 3.1. In the second degradation stage, 1 DTG peak was observed for the hydrogels with varying amounts of ECH (Figure 11a). For the lowest amount of ECH that was added, the DTG peak occurred at 265 °C, higher than the degradation temperatures of GGM and xylan, as seen in Figure 2. Further, a significant amount of GGM was consumed at the lowest ECH level, as evidenced by SEC and the yields (Figure 4 and Figure 7); thus, the rise in temperature was possibly the result of additional bonds (crosslinks), which was also a factor when the ECH was increased from 200 to 250 µL and the DTG peak temperature climbed to 293 °C. However, the LCCs in the solution were bound at higher ECH levels, which also contributed to the increase in DTG peak temperature (Figure 2). The enhancement in yield between 250 and 300 µL ECH was minimal (Figure 4), which explains the small increase in DTG peak temperature from 293 to 298 °C. 

Greater amounts of lignin in the bulk mixture before the reaction had the opposite effect, as apparent from the decrease in DTG peak temperature from 293 to 282 °C in the low-lignin sample and to 271 °C in the high-lignin sample (Figure 11b). This finding is explained by the lower crosslinking, which decreased the amount of bound LCCs, as observed by the change in yields in Figure 5. For the same reason, the results in the previous sections show that the properties of the hydrogel to which the highest amount of lignin was added were similar to that with the lowest ECH content. 

In contrast, the thermal properties of the hydrogels developed a disparate pattern. In addition to the higher DTG peak temperature of 271 °C, an increase in the region of 400 to 600 °C could be observed. The peaks in this region were clearly visible on TGA of the lignin but absent in the GGM sample, as seen in Figure 2, confirming previous indications that the added lignin bound to the hydrogel. This bond was also likely the reason for the increase in temperature for the peak in the region of 200 to 300 °C, wherein lignin had a higher degradation temperature compared with GGM (Figure 2). Although the degradation temperature declined with the addition of lignin, the rate of decomposition decreased as well; thus, the thermal properties of the hydrogels approached those of lignin (Figure 2).

## 4. Conclusions

The combination of ultrafiltration and anti-solvent precipitation was successful in fractionating SSL into 2 streams: polysaccharide-rich and lignin-rich. FTIR measurements of these fractions confirmed the separation of these entities. TGA revealed distinct profiles for GGM and lignin, the latter of which had higher thermal stability. The parameter study showed that the least amount of ECH that was needed for the formation of a hydrogel was 200 µL when 2 mL of stock solution was used. The lignin concentration should also be kept under ~50 g/L to synthesize a coherent gel. 

Based on the variation in the degree of crosslinking, the mannan and glucan were linked, given that their yields during the reactions had the same trend and magnitude. Galactans also followed the same pattern, but their overall yield was low compared with the mannans and glucans, because other galactans existed in the mixture. By LC-MS, the increase in crosslinker concentration accelerated the hydrolysis of ECH to glycerol but also the polymerization of glycerol to di-, tri-, tetra-, penta-, and hexamers. The addition of lignin to the reaction mixture decreased the yields for the polysaccharides and the polymerization of glycerol. However, the consumption of ECH rose, due to the lignin consuming the crosslinker and forming larger water-soluble macromolecules, as seen by LC-MS and SEC. Some of the lignin bound to the hydrogel, as confirmed by the FTIR measurements, wherein the peak intensities for aromatic skeletal vibrations (1509 cm^−1^), guaiacyl structures (1142–1262 cm^−1^), and sulfonate groups (650 cm^−1^) increased.

With regard to swelling degree, hydrogels with low crosslinking reached an equilibrium of approximately 137 g water/g dry hydrogel, and for hydrogels that contained lignin, higher water absorption was observed. The hydrogel with the greatest amount of lignin had the slowest initial release of BTB due to the higher osmotic pressure and water uptake. Repeated swelling and drying of the hydrogels yielded an equilibrium after 2 consecutive cycles, peaking at roughly 50 g/g for hydrogels with the lowest amounts of ECH.

The hydrogels were tested mechanically, based on compression (stress vs strain). A maximum UTS of 21 kPa was reached for the pure GGM hydrogels, whereas the UTS fell with increasing amounts of lignin. A linear relationship between lignin concentration and compression modulus was observed, which also yielded a peak lignin concentration of 40.5 g/L for the formation of a coherent hydrogel. 

The TGA results showed that the degree of crosslinking had a minor effect on the maximum DTG peak temperature; instead, the DTG temperature depended on the yield of the bound LCCs. The addition of lignin to the reaction mixture negatively impacted the DTG peak temperature (decreasing). However, the decomposition rate also declined, increasing the thermal stability of the hydrogel.

## Figures and Tables

**Table 1:** Gradient program for LC of the LC/MS system. Eluent A was 0.1% formic acid solution, and eluent B was 95% acetonitrile in 0.1% formic acid solution. The flow rate was kept constant at 0.5 mL/min.

Time (min)	Eluent A (%)	Eluent B (%)
1	100.0	0.0
8	0.0	100.0
10	0.0	100.0
12	100.0	0.0

**Table 2:** Compositions of the re-dissolved products from the ultrafiltered and anti-solvent precipitated fractions. The samples were dried at 50 °C.

Fraction	TS of Prepared Solutions (g/L)	Lignin Content (g/L)	Acetic Acid (g/L)	Arabinan (g/L)	Galactan (g/L)	Glucan (g/L)	Xylan (g/L)	Mannan (g/L)
GGM	100	8.40	3.60	0.60	15.9	13.0	4.60	23.5
Lignin	99.9	94.6	0.75	0.21	1.09	1.64	0.94	4.11
